# Characteristics of individual *cyp51A* SNPs and combinations thereof impacting the azole phenotype in TR_34_-mediated resistance genotypes of *Aspergillus fumigatus*

**DOI:** 10.1128/aac.01528-25

**Published:** 2026-02-03

**Authors:** Yinggai Song, Jochem B. Buil, Jan Zoll, Marlou Tehupeiory-Kooreman, Hanka Venselaar, Ruoyu Li, Willem J. G. Melchers, Paul E. Verweij

**Affiliations:** 1 Department of Dermatology and Venerology, Peking University First Hospital 26447, Beijing, China; 2 Department of Medical Microbiology, Radboud Institute for Molecular Life Sciences, Radboud University Medical Center, Nijmegen, the Netherlands; 3 Radboudumc-CWZ Center of Expertise for Mycology, Nijmegen, the Netherlands; 4 Research Center for Medical Mycology, Peking University 12465, Beijing, China; 5 Department of Medical BioSciences, Radboudumc, Nijmegen, the Netherlands; University Children's Hospital Münster, Münster, Germany

**Keywords:** *Aspergillus fumigatus*, azole resistant, *cyp51A*, SNPs, molecular docking

## Abstract

The World Health Organization has flagged the rise of drug resistance in *Aspergillus fumigatus* as a critical concern. Elevated mutation rates in this pathogen contribute to the rapid development of resistance, complicating treatment efforts. We conducted a study on the prevalence of azole resistance among clinical *A. fumigatus* isolates in the Netherlands from 1994 to 2022 and identified 34 *cyp51A* variants. To investigate the impact of individual single-nucleotide polymorphisms (SNPs) and combinations thereof on the azole phenotype in TR_34_-mediated resistance genotypes, we focused on novel, recent mutations and explored the effects of SNPs L98H, T289A, I364V, and G448S and the combination of the mutations TR_34_/L98H, TR_34_/L98H/T289A/G448S, and TR_34_/L98H/T289A/I364V/G448S on azole affinity and susceptibility. We created the three-dimensional protein model of the Cyp51A protein with azoles, and the mutation was introduced to the wild-type *cyp51A A. fumigatus* strain by the CRISPR-Cas9 gene editing technique. Finally, *in vitro* susceptibility testing of *A. fumigatus* strains carrying the mutations was conducted to confirm the azole phenotypes observed in clinical isolates. The MICs of all four azoles against the mutated *cyp51A* strains, which harbored combination mutations, were higher than those of the wild type, with highly elevated MICs of itraconazole, voriconazole, and isavuconazole. Genotypes TR_34_/L98H/T289A/I364V/G448S mutant showed a consistent phenotype to the clinical strains, which are highly resistant to voriconazole but susceptible to itraconazole. In this study, we show that molecular dynamics simulations of amino acid substitutions in the *cyp51A* gene correlate to the structure–function relationship of *in vitro* phenotype.

## INTRODUCTION

*Aspergillus fumigatus*, a globally distributed opportunistic pathogen, can cause a broad spectrum of diseases, including invasive aspergillosis (IA), a life-threatening disease in immunocompromised patients (1). Patients at risk for IA include patients with hematologic malignancy, solid organ transplant recipients, and patients receiving corticosteroids. In addition, new risk groups are being recognized, including patients treated with ibrutinib (1) and patients with severe influenza and COVID-19 (2, 3). The fungus might also cause chronic pulmonary infections, chronic lung colonization, and allergic syndromes (4).

Triazole drugs (itraconazole: ITR, voriconazole: VOR, posaconazole: POS, and isavuconazole: ISA) have become the cornerstone of the prevention and treatment of aspergillosis. Unfortunately, azole-resistant *A. fumigatus* (ARAF) has been increasingly found in patients who receive long-term azole treatment, azole-naive patients, and in the agricultural environment (2, 5). The emergence of ARAF isolates has been a significant concern, with increasing resistance rates in some countries (6). The emergence of resistance to azole drugs arises primarily through two genetic mechanisms: point mutations in the *cyp51A* open reading frame (ORF) (with or without tandem repeats [TR_34_/TR_46_] in the promoter region) and overexpression of the *cyp51A* gene. These alterations confer varying levels of resistance to triazole antifungals. Although the TR_34_/L98H and TR_46_/Y121F/T289A are the dominant resistance mechanisms found worldwide, genotype variations have been frequently reported. G54, G138, M220, Y121, G448, and P216 are common hot spot mutations that have been detected in ARAF from patients under long-term antifungal treatment (7, 8). Additionally, resistance can involve upregulation of sterol 14α-demethylase due to *cyp51A* overexpression or reduced intracellular drug concentrations resulting from efflux pump gene overexpression (8–10).

Although resistance might be selected during azole therapy, resistance selection in the environment through exposure to azole fungicides has been shown to be the main route in *A. fumigatus* (11, 12). Point mutations typically emerge from clinical azole exposure (prophylaxis or therapy). In contrast, TR-associated mutations TR_34_/L98H and TR_46_/Y121F/T289A are linked to environmental selection pressure from agricultural azole fungicides, widely used in crop protection (13, 14). These TR strains can disseminate via airborne conidia, infecting azole-naïve patients. Similar trends are observed globally. Two resistant *A. fumigatus* soil isolates harboring mutations of TR_34_/L98H/S297T/F495I were identified in Beijing and Fuzhou, respectively (15). Ren et al. (16) reported the emergence of ARAF carrying the mutations TR_46_/Y121F/T289A and TR_34_/L98H/S297T/F495I in agricultural fields. Multi-azole-resistant strains with these complex mutations have increasingly been isolated in the environment worldwide since 2020. Because there is a concern that their prevalence will likely increase in clinical settings, these strains must continue to be monitored and investigated (17). A novel, multi-azole-resistant *cyp51A* genotype, TR_34_/L98H/T289A/I364V/G448S, was identified in a clinical isolate from the UK. This isolate, recovered in 2016 from a patient with necrotizing aspergillosis, exhibited high MICs for itraconazole and voriconazole (both ≥16 mg/L) and posaconazole (4 mg/L). This specific variant was unique to the clinical case and was not detected in environmental samples (12, 18). Given the threat of environmental and clinical resistance spread—exacerbated by intensive fungicide use (e.g., in Brazil, where pesticide consumption rose 190% by 2010)—monitoring ARAF epidemiology and mechanisms is critical for managing aspergillosis (19).

To elucidate how characteristics of the individual single-nucleotide polymorphisms (SNPs) and combinations thereof impact the azole phenotype within the complex TR_34_/L98H/T289A/I364V/G448S resistance background, this study employed the CRISPR-Cas9 gene editing technique to systematically introduce and evaluate the effects of these specific mutations, both singly and in various combinations.

## RESULTS

### Phenotypes of clinical *A. fumigatus* isolates harboring TR_34_/L98H/T289A/I364V/G448S

The desired mutations were introduced in the *A. fumigatus* A1160∆akuB^ku80^ pyrG^+^ by the CRISPR-Cas9 method (20), which was further utilized for azole susceptibility testing. The recent variant TR_34_/L98H/T289A/I364V/G448S was identified by the 29-year genotype and phenotype screening in our center (Tables S1 and S2; Table 1). The azole profiles of the mutant strains are shown in Table 2. The TR_34_/L98H/T289A/I364V/G448S mutant showed a consistent phenotype for the clinical strains, which are highly resistant to voriconazole but susceptible to itraconazole, and the I364V introduction did not change the phenotype.

**TABLE 1:** TR_34_ variants with triazole phenotypes that are not within the core MIC distribution of TR_34_/L98H *ss* isolates^*a*^

Variant	Isolation date (day/mo/yr)	MIC (mg/L) of:			
		Itraconazole	Voriconazole	Posaconazole	Isavuconazole
TR_34_/L98H *ss*		27.53 (4.78 ± 2.34)	4.87 (2.28 ± 2.95)	0.7 (−0.52 ± 3.18)	7.59 (2.92 ± 2.77)
TR_34_/L98H/S297T/F495I	23/08/2009	>16	**>16**	0.5	**>16**
TR_34_/L98H/S297T/F495I	07/06/2011	>16	4	2	**>16**
TR_34_/L98H/T289A/G448S	18/05/2018	**1**	**>16**	0.25	**>16**
TR_34_/L98H/T289A/G448S	31/01/2020	**1**	**>16**	1	**>16**
TR_34_/L98H/T289A/G448S	04/11/2020	**2**	**>16**	1	**>16**
TR_34_/L98H/T289A/I364V/G448S	27/05/2021	**0.5**	**>16**	0.5	**>16**
TR_34_/L98H/T289A/I364V/G448S	08/06/2021	**2**	**>16**	1	**>16**
TR_34_/L98H/T289A/I364V/G448S	09/08/2021	**1**	**>16**	1	**>16**
TR_34_/L98H/T289A/I364V/G448S	29/09/2021	**1**	**>16**	0.5	**>16**

^*a*^ The abbreviation "*ss*" denotes "*sensu stricto*" and specifies the core group of isolates belonging to the typical T_34_/L98H genotype, distinct from those with additional mutations. For the TR_34_/L98H *sensu stricto* group, values indicate geometric mean MICs, with mean log_2_ MIC ± 3 standard deviations shown in parentheses. Boldface values denote results outside the core MIC distribution.

**TABLE 2:** Susceptibility of medical antifungals and agricultural fungicides^*b*^

Mutant	MIC (mg/L) of:												
	ITR	VOR	POS	ISA	TEB^*a*^	DIF^*a*^	PRO^*a*^	PYR^*a*^	BEN^*a*^	CAR^*a*^	BOS^*a*^	FLUO^*a*^	Olorofim^*a*^
A1160^+^ recipient strain	0.5	0.5	0.12	0.5	0.25	2	0.06	0.016	2	2	16	16	0.016
A1160^+^ cassette *hph*	0.5	0.5	0.12	0.5	0.5	1	0.06	0.06	2	1	16	32	0.03
A1160^+^ L98H	1	1	0.5	2	2	8	0.25	0.03	4	2	16	32	0.03
A1160^+^ T289A	1	0.5	0.12	0.5	0.5	0.5	0.06	0.06	2	1	16	32	0.03
A1160^+^ I364V	0.25	0.5	0.12	0.5	0.5	0.5	0.12	0.06	2	1	16	32	0.016
A1160^+^ G448S	1	8	0.25	4	2	0.12	0.06	0.03	1	1	16	32	0.016
A1160^+^ TR_34_/L98H	>16	4	1	4	4	8	0.06	0.03	1	2	16	32	0.03
A1160^+^ TR_34_/L98H/T289A/G448S	0.5	16	0.25	>16	8	4	4	0.06	2	1	8	16	0.03
A1160^+^ TR_34_/L98H/T289A/I364V/G448S	0.5	>16	1	>16	16	16	4	0.12	2	1	>16	32	0.03

^*a*^ Fungicides read out the MIC_50_.

^*b*^ BEN, benomyl; BOS, boscalid; CAR, carbendazim; DIF, difenoconazole; FLUO, fluopyram; PRO, prochloraz; PYR, pyraclostrobine; TEB, tebuconazole.

Compared to the TR_34_/L98H *sensu stricto* genotype, the TR_34_/L98H/T289A/I364V/G448S variant displayed an inverted resistance profile for itraconazole and voriconazole. We propose that the T289A and G448S mutations are responsible for this change. Furthermore, comparisons with TR_46_/Y121F/T289A/G448S and TR_46_/Y121F/T289A variants (Table S1) indicate that the effects of T289A and G448S are modulated by the genetic context, differing between TR_34_ and TR_46_ backgrounds.

Based on the above phenotype changes, we constructed the important mutants by CRISPR/Cas9 genome editing to explore the mechanism of different mutations: TR_34_/L98H/T289A/I364V/G448S, TR_34_/L98H/T289A/G448S, TR_34_/L98H, L98H, G448S, T289A, and WT *cyp51A*.

### *cyp51A* transformation using CRISPR/Cas9 system

**Fig 1:**
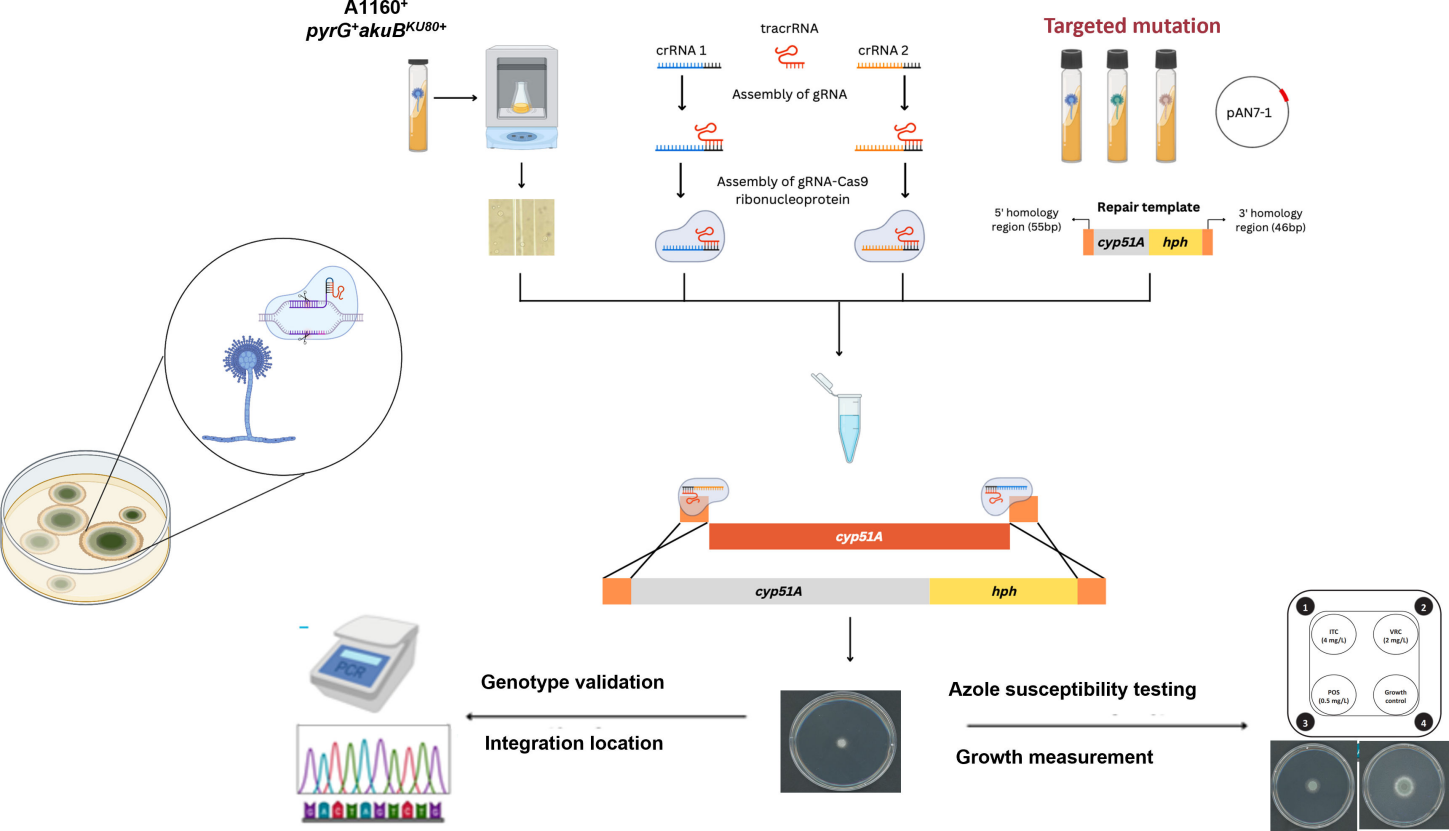
Overview of the microhomology-mediated *cyp51A* transformation using CRISPR-Cas9 gene editing. The overall workflow entails four main sections: (i) strain and culture preparation, (ii) construction of the repair template and the dual Cas9-gRNAs system, (iii) protoplast transformation, and (iv) validation of transformation. *, *P* < 0.05.

In total, three transformations were conducted to create *cyp51A* recombinants of (i) TR_34_/L98H/T289A/I364V/G448S, (ii) TR_34_/L98H/T289A/G448S, (iii) TR_34_/L98H, (iv) L98H, (v) G448S, (vi) T289A, and (vii) WT *cyp51A*, with the selective marker. Several single colonies were chosen from the transformation plates and were sub-cultured on Sabouraud dextrose agar (SDA) slants. By PCR testing, three full combination isolates, three TR_34_/L98H isolates, and three *cyp51A* wild-type and SNP isolates were confirmed to have the recombination occur at the correct location. Subsequently, streaking was performed for all isolates to obtain the pure strains. Sanger sequencing was carried out to validate the locus’s sequence of the successful recombinants (Fig. 1). The insertion of a 34 bp tandem repeat and the substitution of leucine to histidine at position 98 were present in three prospective TR_34_/L98H. Likewise, two wild-type transformants were confirmed to have wild-type *cyp51A* locus. Four desired mutations were successfully introduced in three prospective TR_34_/L98H/T289A/I364V/G448S isolates.

### Baseline susceptibility and single mutations of *A. fumigatus* recombinants

The MICs of four medical azoles—itraconazole, voriconazole, posaconazole, and isavuconazole—against the recombined *A. fumigatus* strains were determined. The wild-type *A. fumigatus* strain (A1160^+^ wt) and its genetically modified control (WT *cyp51A*) show baseline susceptibility to most antifungals (Table 2). Introducing single-point mutations revealed distinct and clinically relevant resistance profiles. The L98H mutation elevated MICs to itraconazole, voriconazole, and isavuconazole, and also resulted in the highest posaconazole MIC (0.5 mg/L) among the single mutants. In contrast, the G448S mutation drove particularly strong resistance to voriconazole (8 mg/L) and isavuconazole (4 mg/L) but had a more moderate effect on posaconazole (0.25 mg/L). Notably, the T289A mutation also substantially increased the itraconazole MIC (1 mg/L) to a level comparable to the G448S mutant, highlighting its previously underestimated role in resistance to this drug. Conversely, the I364V mutation alone did not significantly alter the MIC of any azole, confirming its lack of a direct role in resistance.

### Combined mutations and high-level resistance

The MICs of all four medical azoles against the mutated *cyp51A* strains harboring combination mutations were higher than those of the wild type, with highly elevated MICs of itraconazole, voriconazole, and isavuconazole. Combining mutations generates clinically relevant, high-level multi-azole resistance. The TR₃₄/L98H recombinant exhibits high-level resistance to itraconazole (>16 mg/L) alongside reduced susceptibility to voriconazole, posaconazole, isavuconazole, and tebuconazole. Further combining mutations into the TR₃₄/L98H/T289A/G448S background results in high-level resistance to isavuconazole (>16 mg/L) and voriconazole (16 mg/L), significant resistance to prochloraz (4 mg/L), and reduced susceptibility to other azoles. In the A1160^+^ TR_34_/L98H/T289A/G448S background, the addition of the I364V mutation caused a fourfold increase in the posaconazole MIC, while it did not enhance resistance to itraconazole.

### Non-azole antifungals and olorofim susceptibility

Most non-azole agricultural fungicides retain activity across mutants. The wild-type strain and the majority of mutants, including all single-point mutants, show identical baseline MICs for pyraclostrobine, benomyl, and carbendazim. Fluopyram MICs remain high but stable. Boscalid resistance emerges only in the full combination with I364V mutant. Crucially, the novel antifungal olorofim (dihydroorotate dehydrogenase [DHODH] inhibitor) maintains very low MICs (0.016–0.03 mg/L) against all strains, including the complex pan-azole-resistant mutants (TR₃₄/L98H, full combination with or without I364V), demonstrating no cross-resistance and highlighting its value in managing multi-resistant pathogens.

### High-frequency SNPs G448S and T289A

The two high-frequency hotspot mutations, G448S and T289A, can occur in combination with different genetic backgrounds (TR_34_ or TR_46_). Crucially, they confer distinct, azole-specific phenotypic changes, notably altering the susceptibility profile between itraconazole and voriconazole. Compared to the wild type, the strains with combined *cyp51A* mutations exhibited markedly elevated MICs for voriconazole, posaconazole, and isavuconazole, while the MIC for itraconazole remained at a comparable level (Table 2). The G448S mutants showed high voriconazole (8 mg/L) and isavuconazole (4 mg/L), and high agricultural triazole. While both the T289A and I364V mutants remained phenotypically susceptible to all tested medical and agricultural triazoles according to breakpoints, the T289A mutation consistently resulted in low-level (twofold) MIC elevations for itraconazole and tebuconazole. All mutants are susceptible to olorofim (≤0.03 mg/L) but resistant to fluopyram (≥16 mg/L).

### Phenotype changes vary from mutations

**Fig 2:**
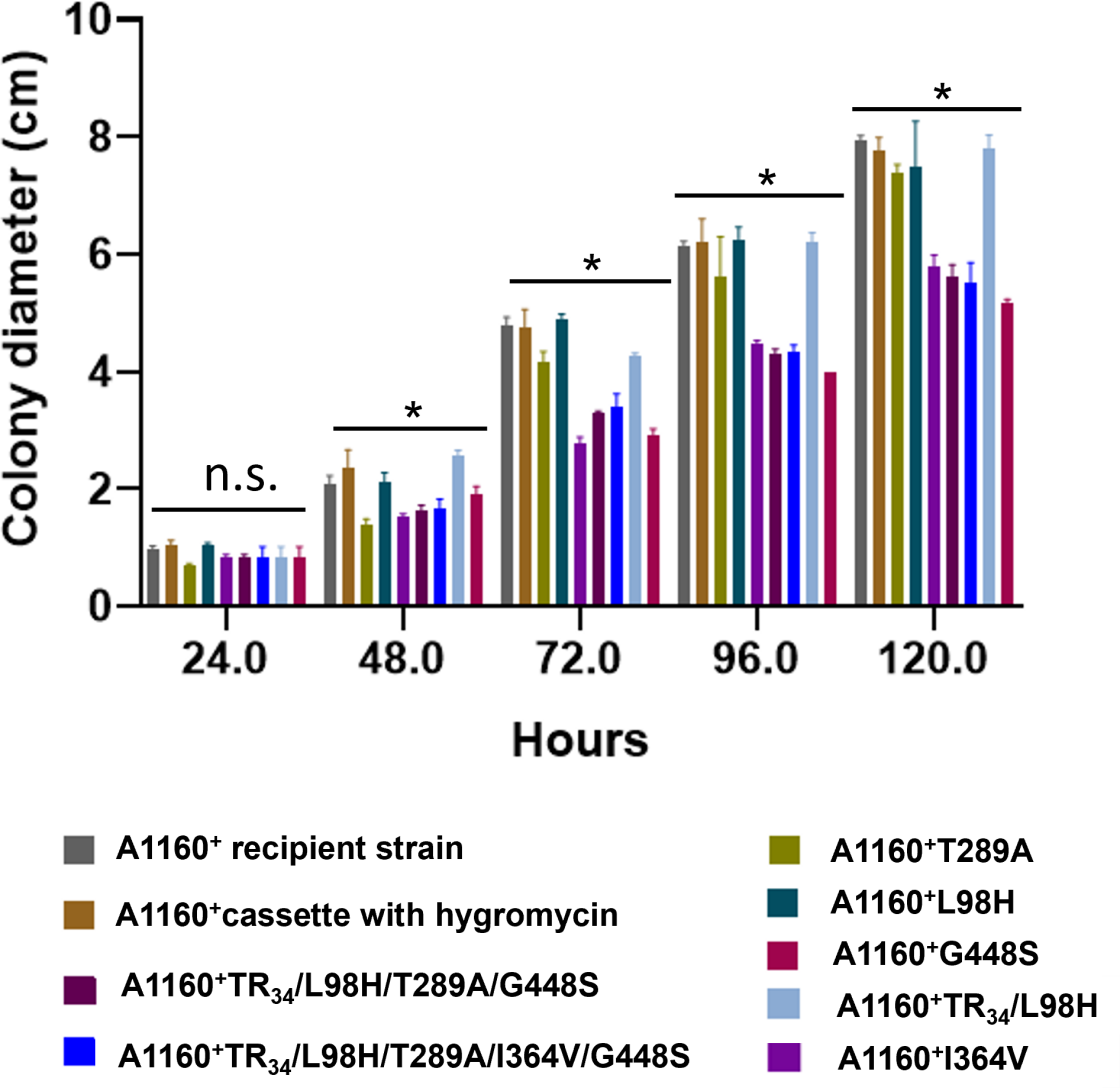
Growth measurements of the transformants. Colony diameter was measured over time to assess the growth rate. The strains analyzed include the wild-type A1160^+^ recipient, a control strain with a hygromycin resistance cassette, and various mutant strains carrying single (T289A, L98H, G448S, and I364V) or combined (TR₃₄/L98H, TR₃₄/L98H/T289A/G448S, and TR₃₄/L98H/T289A/I364V/G448S) resistance-associated mutations.

The degree of resistance varies across different mutations, presumably owing to the intrinsic allele diversity of each isolate. Phenotypic differences between clinical isolates involve many thousands to tens of thousands of SNPs. The average diameter of three independent experiments was used to determine the radial growth rate (Fig. 2). As shown in Fig. 2, several mutant strains, most notably A1160^+^ TR_34_/L98H/T289A/I364V/G448S and A1160^+^ G448S, exhibited statistically significant growth impairment under these conditions. In contrast, control strains (recipient isolate, cassette control) and single mutants (T289A, L98H) show robust, near-normal growth. The double mutant (TR₃₄/L98H) displays only a mild growth reduction, confirming that fitness loss escalates with mutation complexity.

### Molecule docking

**Fig 3:**
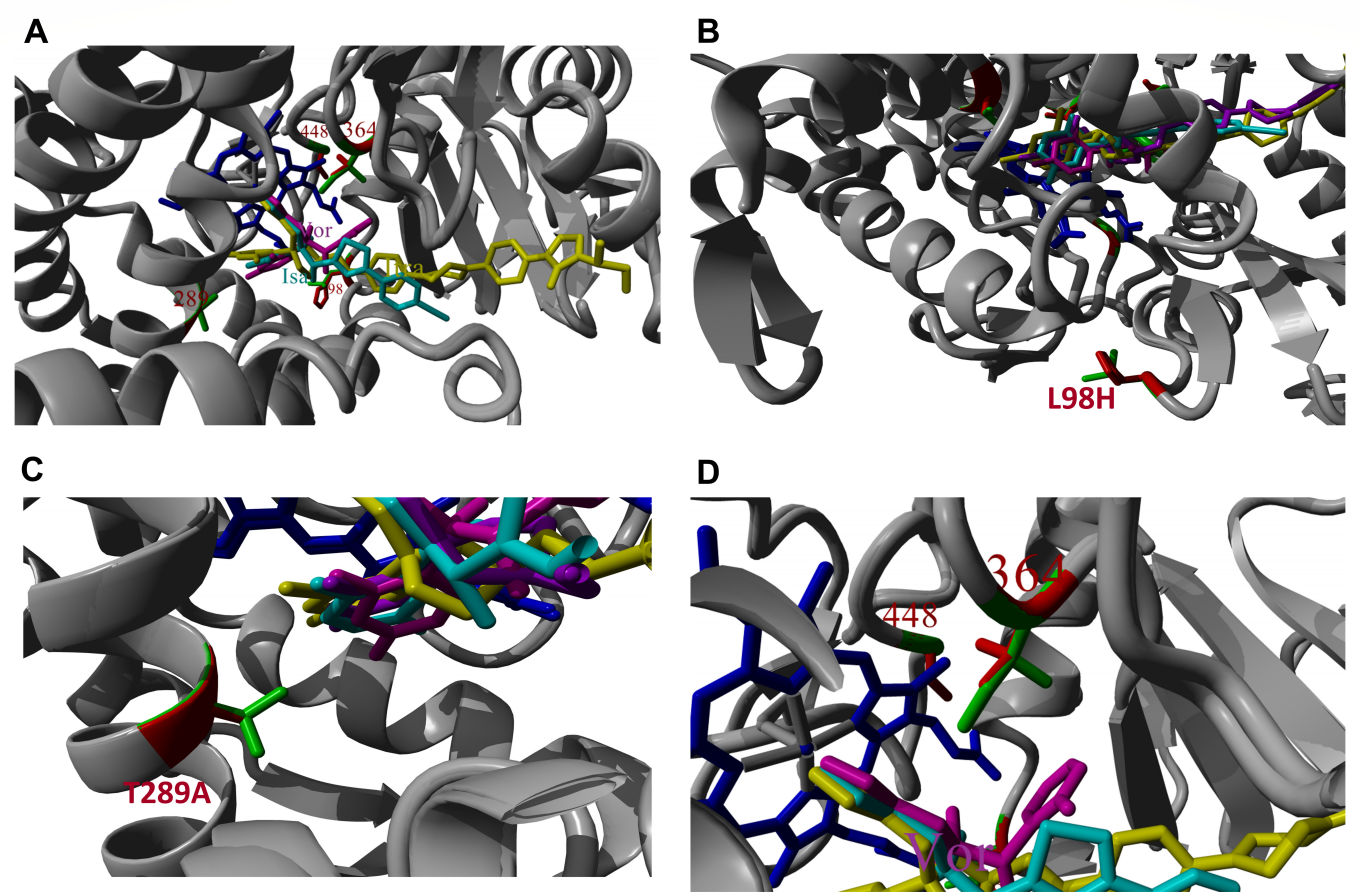
Homology model of *A. fumigatus* Cyp51A. Three-dimensional protein models of the Cyp51A protein with four azoles. The amino acids L98, T289, I364, and G448 are fully exposed and labeled (**A**). Engineered point mutations at critical residues (L98H, T289A, I364V, and G448S), TR_34_/L98H/T289A/I364V/G448S, are fully solvent-exposed and labeled (**B, C, D**). These models show the precise location of key residues (L98, T289, I364, and G448) and the conformational changes induced by the mutations. Red, isavuconazole; yellow, itraconazole; purple, posaconazole; and magenta, voriconazole.

Molecular dynamics simulations form a systematic atlas that pairs wild-type Cyp51A snapshots with close-up views of key resistance mutations found in two multi-azole-resistant *A. fumigatus* haplotypes. They are arranged so that each set of “close-up” panels highlights the exact residue, side-chain orientation, and local pocket changes introduced by the mutations. To understand how the combination of mutations in the TR_34_/L98H backbone leads to resistance against all medical azoles, we performed molecular docking simulations. Figure 3A presents a simplified model of these findings. The L98H mutation destabilizes heme binding while increasing BC-loop flexibility, narrowing the ligand entry channel (Fig. 3B). T289A disrupts hydrogen-bond networks near the heme, compromising triazole coordination (Fig. 3C). G448S creates steric hindrance at the substrate channel entrance, blocking bulky azoles (isavuconazole). I364V compresses the hydrophobic cavity, restricting accommodation of hydrophobicity-dependent azoles (voriconazole/posaconazole) (Fig. 3D). These mutations form a complementary defense barrier—L98H and T289A target the catalytic core, G448S guards the channel gateway, and I364V remodels the binding cavity—collectively dismantling the binding foundations of diverse azoles. The combinatorial mutations in *A. fumigatus* Cyp51A (L98H/T289A/I364V/G448S) confer pan-azole resistance through precise spatial reorganization. Despite these resistance-conferring changes, lanosterol (natural substrate) maintains binding through (i) conformational flexibility of the BC loop, compensating for mutation-induced distortions; (ii) minimal impact of T289A/L98H on substrate orientation; and (iii) tolerated geometry shifts at G448S/I364V due to hydrophobic stacking adaptability. This functional duality—simultaneous azole resistance and sustained lanosterol 14α-demethylation—explains both the high MICs (>16 mg/L) observed clinically and the environmental fitness of TR_34_-harboring strains in fungicide-exposed niches.

## DISCUSSION

Drug-resistant fungal infections are a major concern and challenge for the medicines and agriculture (21). Resistance in *A. fumigatus* is linked to the accumulation of agricultural azoles in its natural habitats, such as decaying plant material, which amplifies resistant genotypes. Cross-resistance between agricultural azoles (e.g., tebuconazole) and medical azoles (e.g., voriconazole) complicates treatment, with resistant isolates often harboring signature mutations like TR_34_/L98H and TR_46_/Y121F/T289A (22). Genomic studies confirm overlap between environmental and clinical-resistant populations, highlighting the role of agricultural practices in clinical resistance (12).

The CRISPR-engineered *cyp51A* mutants reveal a synergistic resistance mechanism of combinatorial mutations in *A. fumigatus*. In this study, we employed the CRISPR-Cas9 gene editing technique to investigate the effects of individual SNPs and combinations thereof on the azole phenotype in TR_34_/L98H/T289A/I364V/G448S-mediated resistance genotypes. Our CRISPR-Cas9 mutagenesis reveals that G448S is the primary driver of voriconazole and isavuconazole resistance in single-mutant *A. fumigatus* strains. Combinatorial mutations within the TR_34_/L98H backbone, however, induce synergistic high-level resistance. The TR_34_/L98H/T289A/G448S confers MIC > 16 mg/L for VOR/ISA, while adding I364V (TR_34_/L98H/T289A/I364V/G448S) amplifies pan-azole resistance. Structural analyses explain these profiles: the G448S mutation introduces a steric barrier at the channel entrance that preferentially hinders the passage of triazoles with bulky, rigid tail groups, such as voriconazole and isavuconazole. Concurrently, the I364V mutation contracts the hydrophobic cavity, primarily affecting the binding of posaconazole. The T289A mutation disrupts hydrogen-bond networks critical for anchoring smaller triazoles, while L98H impairs heme-binding stability. Notably, the I364V mutation exerts a context-dependent effect on posaconazole resistance. While it alone did not alter susceptibility, it caused a fourfold MIC increase when introduced into the TR_34_/L98H/T289A/G448S background. This suggests that the combined T289A and G448S mutations pre-configure the Cyp51A structure, making it susceptible to a subtle but critical steric shift induced by I364V, which specifically compromises posaconazole binding. This epistatic interaction highlights how resistance evolves through the synergistic effect of multiple mutations.

Furthermore, we found that the high-level itraconazole resistance seen in TR_34_/L98H was substantially attenuated in strains carrying additional mutations like T289A and G448S. This suggests that the Cyp51A binding pocket is highly tunable. We speculate that the introduction of these secondary mutations reconfigures the pocket’s topology, potentially inducing a steric clash that specifically compromises itraconazole binding. This represents a potential evolutionary trade-off, where the fungus optimizes resistance toward one azole at the expense of another, highlighting the complex pathways of antifungal resistance evolution. Our findings directly support the establishment of a national surveillance network to monitor trends in the frequency of azole resistance in *A. fumigatus* and to better understand the underlying resistance mechanisms.

Critically, the fungus maintains essential lanosterol 14α-demethylase activity through compensatory mechanisms. This may involve functional redundancy from the paralogous Cyp51B enzyme, which could partially offset any impairment in the mutated Cyp51A, alongside minimal disruption of the Cyp51A active site itself. Thus, by ensuring uninterrupted membrane biosynthesis while effectively excluding drugs, these mutations achieve a sophisticated equilibrium between pan-resistance and ecological fitness. The clinical resistance patterns showed limited extension to the tested agricultural fungicides. All mutants displayed reduced susceptibility to fluopyram (MIC: 16–32 mg/L), while remaining susceptible to carbendazim (MIC: 1–2 mg/L) (Table 2). Notably, cross-resistance within the succinate dehydrogenase inhibitors (SDHIs) class emerged specifically in combinatorial mutants, suggesting that the complex cyp51A haplotypes can confer broader fungicide tolerance.

A key finding of this study is the restored susceptibility to itraconazole observed in strains carrying additional T289A and G448S mutations alongside the resistant TR_34_/L98H background. We hypothesize that this phenomenon may be due to a conformational change in the Cyp51A enzyme, induced by the combination of T289A and G448S, which remodels the itraconazole-binding pocket and reduces drug affinity. Other potential mechanisms include compromised protein stability or disrupted interactions with the heme cofactor, indirectly impairing binding. Furthermore, these mutations may confer a general fitness cost or alter membrane integrity, rendering the strain more vulnerable to azole stress.

Despite increased *A. fumigatus* isolation during the COVID-19 pandemic, azole resistance rates remained stable—a phenomenon potentially reflecting diagnostic biases or ecological equilibria in TR_34_ genotype distribution. While azoles remain first-line therapy, combinatorial resistance undermines efficacy: high-dose isavuconazole shows limited utility against G448S strains, and co-infections (azole-susceptible + resistant strains) complicate diagnostics. The accumulation of diverse *cyp51A* mutations over time drives fluctuating resistance patterns, making treatment and antifungal stewardship challenging. Current culture/PCR methods fail to detect cryptic SNPs and heteroresistance, underscoring the urgent need for NGS-based diagnostic panels and monitoring air/soil/clinical matrices using molecular tools (23, 24).

Clinical isolates harbor complex resistance mutations. Diversity mutations of *cyp51A* accumulated over time and resistant variants in clinical isolates are related to variation in the environment. The spectrum of azole resistance is driven by diverse genetic mutations and is influenced by treatment practices. This complexity, in turn, poses significant challenges for effective antifungal stewardship and clinical management. Compensatory evolution through mutations allows resistant *A. fumigatus* to overcome potential fitness costs. The presence and clinical implications of compensatory evolution in *A. fumigatus* are largely unknown. Multidisciplinary and intersectoral collaborations are needed to counter the spread of antifungal resistance, diagnostic stewardship in healthcare, and antifungal stewardship in the environment.

This study moves beyond correlation to establish causality and to quantify the individual and interactive roles of key mutations within a new variant, multi-drug-resistant fungal genotype, and illustrates how specific *cyp51A* mutations confer targeted azole resistance, while combinations involving the TR₃₄ promoter and multiple mutations drive broad, high-level pan-azole resistance, mirroring challenging clinical isolates. The sustained potent activity of olorofim against all resistant strains highlights its distinct mechanism of action and significant therapeutic potential for treating multi-drug-resistant *A. fumigatus* infections. It provides fundamental mechanistic knowledge crucial for combating the growing threat of azole-resistant fungal infections through improved diagnostics, drug discovery, and treatment strategies.

## MATERIALS AND METHODS

### Screening of azole-resistant *A. fumigatus* clinical isolates

We collected 12,679 clinical *A. fumigatus* isolates from 1994 to 2022, preserved at Radboud University Medical Centre. MIC testing was performed according to the European Committee on Antimicrobial Susceptibility Testing (EUCAST) broth microdilution reference method (25) for amphotericin B (AmB), itraconazole, voriconazole, posaconazole, and isavuconazole (from 2015 when the drug was clinically licensed). An agar plate containing only RPMI 1640 with 2% glucose agar was used as a growth control. Triazoles with activity against *A. fumigatus* include itraconazole, voriconazole, posaconazole, and isavuconazole. Azole resistance was defined as resistance to >1 azole drug, according to EUCAST clinical breakpoints (itraconazole, >2 mg/L; voriconazole, >2 mg/L; posaconazole, >0.25 mg/L, and isavuconazole, >1 mg/L). EUCAST broth microdilution plates were made at Radboud UMC in batches of 96-well plates and complied with the recommended quality control standards.

### Isolates with resistance genotype and phenotype

For four *A. fumigatus* isolates with a confirmed azole-resistant phenotype, the full c*yp51A* gene was analyzed by PCR amplification and sequencing. The c*yp51A* sequence (GenBank accession no. AF338659) was used for mutation analysis. A spore suspension of all isolates was stored at –80°C in 10% glycerol. Clinical information regarding the underlying disease and classification of *Aspergillus* disease was not collected.

### CRISPR/Cas9 lab strain and culture preparation

The parental strain used in this experiment was the *cyp51A* wild-type *A. fumigatus* A1160∆akuB^ku80^ pyrG^+^, a derivative of the CEA10 clinical lineage which lacks a nonhomologous end-joining DNA repair pathway (20). This strain has been commonly employed in studies that performed genetic manipulation by integrating exogenous DNA using homologous recombination. The parental strain was taken from the freezer stock of the Radboudumc Mycology Research Group and cultured twice on SDA (Oxoid) media to activate sporulation. The desired cyp51A wild-type genotype was confirmed by sequencing. A new culture from the freezer stock was done after every three transformations.

### Construction of the repair template and the dual Cas9-gRNAs system

All primer sequences and crRNA designs are listed in Table 3.

**TABLE 3:** Sequences of primers and crRNAs used in the CRISPR/Cas9 system

Name	Sequence
cyp-F 5′	5′-GGCTTTCATATGTTGCTCAGCGGCAGCATTCTGAAACACGTGCGTAGCAAGCGAGAAGGAAAGAAGCAC
cyp-R 3′	5′-GGTCTGAATAAGGGTTCAATACAGTCATTTATTAGGCGCTCGAGGGGCTGAATTAAGTATAA
hyg-F	5′-TATTGAACCCTTATTCAGACCACGGCGTAACCAAAAGTCACACAACACAAGCTGTAAG
hyg-R	5′-CAAATACTCATACTCAGTATAGGCAACAACACTTCAGGGCCAGTAATCTTGACGACCGTTGATCTG
fusion-F	5′-TATTGAACCCTTATTCAGACCACGGCGTAACCAAAAGTCACACAACACAAGCTGTAAG
fusion-R	5′-CAAATACTCATACTCAGTATAGGCAACAACACTTCAGGGCCAGTAATCTTGACGACCGTTGATCTG
hyg-cyp51-F	5′-GGCTTTCATATGTTGCTCAGCG
hyg-cyp51-R	5′-CAAATACTCATACTCAGTATAGGCAACAACAC
5′ gRNA	TCTGAAACACGTGCGTAGCA
3′ gRNA	ATACTTAATTCAGCCCCTCG

The hygromycin B phosphotransferase (*hph*) cassette was integrated into the 3′-UTR of the *cyp51A* repair template, using a 117 bp sequence from the ORF as the homologous arm for Cas9-mediated gene replacement. The correct integration and orientation of the cassette were verified by DNA sequencing. Furthermore, RNA-seq analysis confirmed that this integration did not disrupt the transcription of the *cyp51A* gene itself or its adjacent genes. A 2,695 bp *hph* sequence was amplified from pAN7-1 plasmid with hyg-F and hyg-R primers using PCR. The plasmid remnants were digested by the DpnI enzyme (New England BioLabs) (Fig. S1).

The *cyp51A* repair templates with four desired *cyp51A* genotypes (i) wild-type cyp51A, (ii) TR_34_/L98H, (iii) TR_34_/L98H/T289A/G448S, and (iv) TR_34_/L98H/T289A/I364V/G448S were isolated from the clinical strains. These genotypes were first confirmed by Sanger sequencing before the PCR amplification step. The primers used for the *cyp51A* amplification were designated as cyp-F 5′ and cyp-R 3′, targeting the 5′-UTR and 3′-UTR, respectively (Table 3). cyp-R 3′ and hygF shared a 21 bp overlapping sequence, which would function as a linker for the fusion of the *cyp51A* and the *hph* cassette. The hyg-R primer was designed to insert 46 bp of flanking microhomology region for the cyp51A 3′-UTR. The resulting *hph* and *cyp51A* fragments were purified using QIAquick Gel Purification Kit (Thermo Fisher Scientific) and fused by Gibson Assembly Master Mix. The fused fragments were further amplified by PCR using a shorter set of primers: fusion-F and fusion-R. The final PCR products were purified and utilized as repair templates.

We replaced the parental *cyp51A* gene with the desired *cyp51A* genotypes. To knock out the whole ORF and the prospective tandem repeat region, two crRNAs targeting the 5′-UTR and the 3′-UTR of the *cyp51A* gene were designed using the web-based guide RNA designing tool EuPaGDT (26). The genomic sequence of the 5′-UTR and the 3′-UTR from the genomic database was then uploaded to the EuPaGDT website. The program was run with the SpCas9 selection, microhomology search, and conserved region search settings. The generated crRNA would be chosen if it was close to the ORF and had the highest QC score. Alt-R CRISPR-Cas9 crRNA (2 nmol), Alt-R CRISPR-Cas9 tracRNA (5 nmol), and Alt-R s.p. Cas9-nuclease (100 μg) were ordered from Integrated DNA Technology (IDT). A 100 μM stock solution of the crRNA and tracRNA each was made by resuspending the RNA oligos in a nuclease-free duplex buffer supplied by the IDT. For each gRNA (3′ gRNA and 5′ gRNA), a 33 µM RNA duplex solution was prepared by mixing an equimolar concentration of the crRNA and tracRNA in the nuclease-free duplex buffer. The solution was then heated at 95°C for 5 min and cooled to room temperature gradually in a thermal cycler for 10 min (0.1°C/s). The Cas9-nuclease was diluted 10 times in the Cas9 working buffer (20 mM HEPES; 150 mM KCl, pH 7.5) to reach a final concentration of 1 µg/µL. All the prepared molecular reagents were stored at −20°C.

### Protoplast transformation

The procedure was established mostly based on the methodology of Zhao et al. (20). Approximately 1 × 10^6^ fresh conidia of the parental *A. fumigatus* were inoculated in 50 mL Sabouraud dextrose broth (Thermo Fisher Scientific) at 37°C with shaking at 130 rpm. After 16 h, the mycelia were harvested by filtration through sterile Miracloth and resuspended in 20 mL yeast extract glucose solution (YG; 0.5% yeast extract, 2% glucose) with protoplast solution (2 g VinoTaste Pro [Lamothe-Abiet] in 20 mL 1 M KCl and 0.1 M citric acid). After approximately 2 h, the protoplasts were checked for proper size and harvested through a sterile Miracloth filter to discard the mycelial debris. The protoplast solution was centrifuged for 10 min at 2,000 × *g* at 4°C. Following discarding the supernatant, the protoplast pellet was washed twice and resuspended in 0.6 M KCl and 50 mM CaCl_2_. The protoplast solution was kept on ice before use. Five minutes before the transformation, the Cas9:crRNA:tracRNA ribonucleotide complex (RNP) was assembled by mixing 1.5 µL of the 3′ gRNA, 5′ gRNA, and Cas9-nuclease (1 µg/µL) in 22 µL Cas9 working buffer (total volume of 26.5 µL). After 5 min of incubation at room temperature, the RNP complex solution, 20 µL dsDNA repair template, and 25 µL PEG solution (60% wt/vol PEG3350, 50 mM CaCl_2_, 450 mM Tris-HCl, pH 7.5) were added to 50 µL protoplast solution. The mixture was incubated on ice for 50 min before supplementing an additional 1 mL PEG solution, followed by another 25 min of incubation at room temperature. The entire transformation solution was plated over five SMM agar plates (sorbitol minimal medium [SMM]: glucose minimal medium [GMM] supplemented with 1 M sorbitol and 1.5% [wt/vol] agar) containing hygromycin B (150 µg/mL). The plates were incubated at room temperature for the first 24 h and then moved to the 37°C incubator for 48–72 h.

### Validation of CRISPR/Cas9 transformants

Single colonies on SMM agar plates supplemented with hygromycin B were selected and transferred to SDA (Thermo Fisher Scientific). After 2 days of incubation at 37°C, the conidia were collected for DNA extraction. The conidia were first suspended in 250 µL breaking buffer (2% Triton X-200, 1% SDS, 100 mM NaCl, 10 mM Tris-HCl, pH = 8, 1 mM EDTA, pH = 8) and shaken with glass beads (0.4–0.6 mm diameter) at maximum speed for 30 min at 70°C. Afterward, 200 µL phenol-chloroform-isoamyl alcohol was added, followed by a 5 min shake at maximum speed at room temperature. The mixture was centrifuged for 5 min at 11,000 × *g*. The upper phase containing the DNA was collected. The recombinant isolates were first checked for the position of integration. To check whether the repair template was integrated into the correct position, a set of two primers designated as hyg-cyp51-F and hyg-cyp51-R was used, which covered an upstream region of the cyp51A 5′-UTR and a part of the *hph* cassette, respectively. If the recombination happened correctly, a PCR product of around 3 kb would be obtained. Isolates with correct integration positions had their *cyp51A* sequenced by Sanger sequencing to validate the genotypes of interest. The phenotypic stability and Mendelian segregation of the introduced mutations in subsequent generations were consistent with a single, precise genetic modification at the native *cyp51A* locus.

### Growth testing and antifungal susceptibility testing of *A. fumigatus* recombinants

Growth measurements of the recombinants compared to the transformation recipient isolate and the transformation control cassette isolate. The average diameter of three independent experiments was used to determine the radial growth rate. The azole phenotype was characterized by the MIC of the medical azole compounds against *A. fumigatus*, which was determined according to the EUCAST E.Def 9.4 methodology for broth microdilution susceptibility testing; the novel antifungal olorofim and the fungicide susceptibility testing were performed against tebuconazole, difenoconazole, prochloraz, pyraclostrobine, benomyl, carbendazim, boscalid, and fluopyram. We also conducted testing for fungicides on the recombinants, covering five distinct targets, including agricultural triazole, imidazole, quinone outside inhibitors, methyl benzimidazole carbamate, and SDHI.

### Three-dimensional protein model of the Cyp51A protein with azoles

Since no experimentally solved 3D structure for *A. fumigatus* Cyp51A exists, a homology model using the YASARA & WHAT IF Twinset software was created (27, 28). The standard YASARA modeling protocol with default settings was used. As possible templates, the previously solved structures were chosen: *A. fumigatus* Cyp51B (PDB file 6CR2, 64% sequence identity) and *Saccharomyces cerevisiae* Cyp51 (PDB files 5ESM and 8DL4, 49% sequence identity). The best parts of the created models were selected by the automatic modeling script to create a final hybrid model that was used for further analysis. The azole structures used in this study were obtained from PDB files 4UYM (voriconazole), 5ESL (itraconazole), 6E8R (posaconazole), and 6UX0 (isavuconazole). These molecules were superposed on the VNI-derivative molecule present in the hybrid Cyp51 model, followed by an energy minimization run using the standard YASARA script. The four different azole/protein combinations were compared and analyzed.

This 3D homology model visualizes *A. fumigatus* Cyp51A, the target of medical triazoles, with engineered point mutations at critical residues (L98, T289, I364, and G448) fully solvent-exposed and labeled—key sites for azole resistance (e.g., TR_34_/L98H). The substrate access channels and binding regions are indicated in the structural diagram (Fig. 3), revealing steric constraints for antifungal drugs. Experimentally docked azole ligands include itraconazole (visible, 4,167 atoms) and voriconazole (visible, 12,640 atoms), while isavuconazole and posaconazole are inactive. Real-time structural metrics are enabled for bond lengths, angles, and dihedrals, facilitating analysis of mutation-induced conformational changes in substrate channels and ligand-binding efficiency. This model directly correlates residue exposure with clinical resistance mechanisms, providing a mechanistic basis for azole treatment failures.

## SUPPLEMENTAL MATERIAL

Supplemental material aac.01528-25-s0001.pdf Fig. S1; Tables S1 and S2.

ASM does not own the copyrights to Supplemental Material that may be linked to, or accessed through, an article. The authors have granted ASM a non-exclusive, world-wide license to publish the Supplemental Material files. Please contact the corresponding author directly for reuse.
