# Supplementary material for: Characteristics of individual cyp51A SNPs and combinations thereof impacting the azole phenotype in TR34-mediated resistance genotypes of Aspergillus fumigatus
Source: Antimicrob Agents Chemother. 2026 Feb 3;70(3):e01528-25. doi: 10.1128/aac.01528-25 (PMC12959090; doi:10.1128/aac.01528-25)
Supplement: Supplemental material — Fig. S1; Tables S1 and S2. [file aac.01528-25-s0001.pdf]

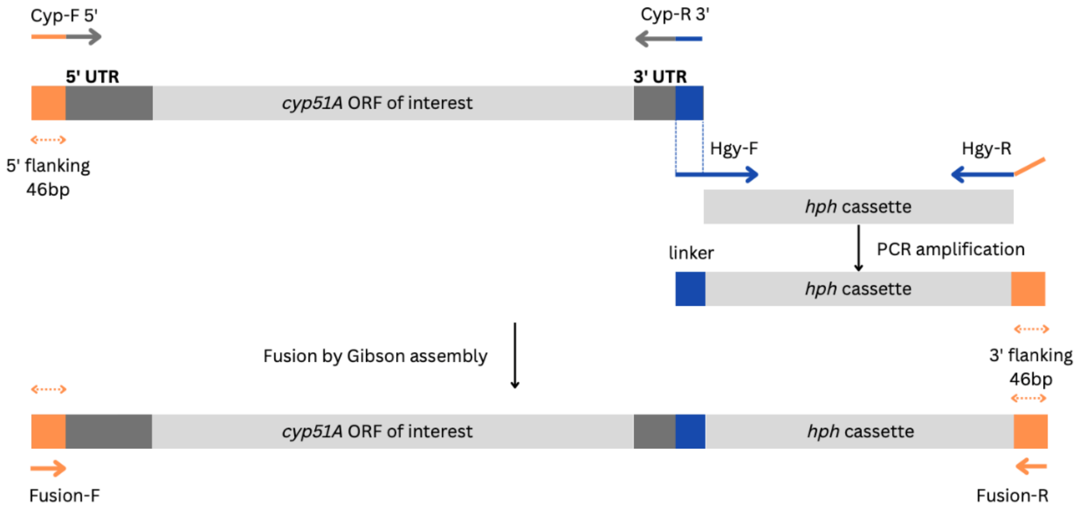

Figure S1: Schematic overview of the repair template construct.

The hph cassette was added in the 3'-UTR of the *cyp51A* gene. The cyp51A fragment was amplified with primers cyp-F 5' and cyp-R 3'. The hph cassette was amplified from the pAN7-1 plasmid using hyg-R and hyg-F primers. Primers cyp-R 3' and hyg-F were designed to share an overlapping sequence, serving as a linker for the fusion of *cyp51A* template and hph cassette. fusion-F and fusion-R were used to amplify the final repair templates.

Table S1 The antifungal susceptibility overview of *Aspergillus fumigatus*

The table data illustrate the distribution of azole MICs across different genetic backgrounds, confirming the multi-azole resistance conferred by the TR$_{34}$/L98H genotype.

**Genotype (N)**	**Itraconazole MIC (mg/L)**			**Voriconazole MIC (mg/L)**			**Posaconazole MIC (mg/L)**			**Isavuconazole MIC (mg/L)**		
	GM	MIC$_{50}$	Range	GM	MIC$_{50}$	Range	GM	MIC$_{50}$	Range	GM	MIC$_{50}$	Range
**TR$_{34}$/L98H (1621)**	6.46	16	0.125-64	6.02	8	0.25->16	0.25	0.5	0.002->16	6.03	8	0.5->16
**TR$_{46}$/Y121F/T289A (321)**	2.807	2	0.25->64	13.71	16	8->64	0.288	0.5	0.002->8	12.179	16	4->16
**TR$_{34}$/T-67G/L98H (16)**	8.354	16	1->16	2.828	2	2-8	0.5	0.25	0.125-1	3.513	4	2-8
**TR$_{34}$/L98H/S297T/F495I (38)**	12.39	16	0.5->16	2.4	2	0.5->8	0.51	0.5	0.008-16	16	16	>=16
**TR$_{34}$/L98H/T289A/I364V/G448S (5)**	1.74	1	0.5->16	16	16	>16	0.76	1	0.5-1	16	16	>16
**TR$_{46}$/Y121F/M172I/T289A/G448S (9)**	9.332	16	1->16	11.758	16	4->16	0.250	0.125	0.063-2	2.333	2	0.25->16
**TR$_{46}$/Y121F/T289A/S363P/I364V/G448S (33)**	6.088	16	0.5->16	15.023	16	8->16	0.959	1	0.5-4	15.667	16	8->16
**TR$_{46}$/Y121F/T289A/G448S (11)**	2.573	1	0.5->16	13.244	16	8->16	0.828	1	0.12-16	11.676	16	8->16

**TR_{92}/Y121F/M172I/T289A/G448S (14)**	4.876	4	0.5->16	12.491	16	2->16	0.609	0.5	0.031->16	11.314	16	2->16

Note: GM: Geometric Mean of the MIC values. MIC_{50}: The Minimum Inhibitory Concentration at which 50% of the isolates are inhibited. T-67G: A nucleotide substitution (T→G) at position 67 in the *cyp51A* promoter region.

Table S2 The antifungal susceptibility of *Aspergillus fumigatus* isolated from each year

**Genotype**	**Itraconazole MIC*(mg/L)**			**Voriconazole MIC*(mg/L)**			**Posaconazole MIC*(mg/L)**			**Isavuconazole MIC*(mg/L)**		
**(N)**	GM	MIC_{50}	Range	GM	MIC_{50}	Range	GM	MIC_{50}	Range	GM	MIC_{50}	Range
**TR_{34}/L98H**												
**2021(n=70)**	13.52	16	0.25->16	4.97	4	0.5->16	0.80	1	0.125->8	7.17	8	0.5->16
**2020(n=83)**	15.22	16	2->16	3.84	4	2-16	0.64	0.5	0.25-4	5.97	4	0.5-16
**2019(n=127)**	14.11	16	2->16	3.73	4	2-8	0.46	0.5	0.12-2	5.75	8	4->16

**2018(n=149)**	14.17	16	0.25->16	3.17	4	0.5->16	0.61	0.5	0.063-16	5.75	8	0.5->16
**2017(n=150)**	14.93	16	2->16	3.61	4	2->8	0.75	1	0.125->8	6.12	8	1->16
**2016 (n=114)**	0.89	1	0.25-8	9.54	8	4->16	0.01	0.008	0.002-0.125			
**2015 (n=93)**	0.76	1	0.25-1	10.54	8	4->16	0.01	0.008	0.002-1			
**2014 (n=52)**	4	16	0.25->16	6.82	8	4->16	0.15	0.5	0.016->16			
**2013 (n=60)**	16	16	>16	7.20	8	2->16	1.03	1	0.25->16			
**2012 (n=39)**	15.17	16	4->16	8	8	2->16	1.13	1	0.25-8			
**2011 (n=52)**	14.38	16	1->16	7.69	8	2->16	0.83	1	0.031-2			
**2010 (n=70)**	16.32	16	16-64	6.50	8	2-16	1.15	1	0.25->16			
**2009 (n=66)**	14.56	16	0.125->64	5.26	4	1->16	0.35	0.25	0.06-1			
**2008 (n=20)**	16	16	>16	8.88	8	2-16	0.37	0.5	0.016-1			
**2007 (n=24)**	12.70	16	0.125->16	5.99	8	0.25-16	0.55	0.5	0.031-2			
**2006 (n=13)**	11.02	16	2->16	5.81	4	4-8	0.38	0.5	0.25-0.5			

**2005 (n=8)**	14.67	16	8->16	3.36	4	0.5-16	0.5	0.5	0.12-1			
**2004 (n=10)**	11.31	16	4->16	4	4	4	0.35	0.5	0.25-0.5			
**2002 (n=6)**	16	16	>16	4.49	4	0.5-1	0.63	0.5	2-8			
**TR$_{46}$/Y121F/T289A**												
**2021 (n=18)**	2.25	1	0.25->16	16	16	>16	0.5	0.5	0.25-2	16	16	>16
**2020 (n=28)**	5.12	4	0.25->16	16	16	>16	0.54	0.5	0.25-2	16	16	>16
**2019 (n=23)**	2.87	2	0.5->16	16	16	>16	0.37	0.5	0.12-1	10.81	8	8->16
**2018 (n=22)**	2	2	0.25->16	8.79	8	8->16	0.57	0.5	0.12-1	15.02	16	4->16
**2017 (n=35)**	3.41	2	0.5->16	8	8	>8	0.85	1	0.25->8	8	8	4->16
**2016 (n=26)**	0.71	0.5	0.25-2	10.73	8	8-16	0.01	0.008	0.002-0.125			
**2015 (n= 22)**	0.5	0.5	0.25-1	16	16	>16	0.01	0.004	0.002-0.031			
**2014 (n=36)**	1.82	1	0.25->16	14.75	16	4->16	0.14	0.25	0.004-2			
**2013 (n=23)**	6.68	16	0.5->16	16	16	>16	0.79	1	0.25-2			

**2012 (n=27)**	10.61	16	1->16	16	16	>16	0.93	1	0.5-4
**2011 (n=22)**	16	16	1->16	17.15	16	2->16	0.84	1	0.031-2
**2010 (n=40)**	1.90	1	0.25->16	16	16	>16	0.5	0.25	0.25-4
